# Occurrence and Antimicrobial Susceptibility Profile of *Salmonella* Isolates from Animal Origin Food Items in Selected Areas of Arsi Zone, Southeastern Ethiopia, 2018/19

**DOI:** 10.1155/2021/6633522

**Published:** 2021-03-31

**Authors:** Minda Asfaw Geresu, Behailu Assefa Wayuo, Gezahegne Mamo Kassa

**Affiliations:** ^1^Department of Veterinary Science, College of Agriculture and Environmental Science, Arsi University, Asella, Ethiopia; ^2^College of Veterinary Medicine and Agriculture, Addis Ababa University, Bishoftu, Ethiopia

## Abstract

The status of *Salmonella* and its antimicrobial susceptibility profile in animal origin food items from different catering establishments in Ethiopia is scarce. Hence, this study aimed to investigate the occurrence and antimicrobial susceptibility profile of *Salmonella* isolates from animal origin food items in the selected areas of Arsi Zone. One hundred ninety-two animal origin food samples were collected and processed for *Salmonella* isolation. Isolates were tested for their susceptibility to 13 antimicrobials using Kirby–Bauer disk diffusion assay. An overall prevalence of 9.4% (18/192) *Salmonella* spp. isolates were recovered from animal origin food samples collected from different catering establishments. Seven (21.9%) of *“Dulet,”* 4 (12.5%) of *“Kitfo,”* 3 (9.4%) of *“Kurt,”* 2 (6.3%) of raw milk, 1 (3.1%) of egg sandwich and 1 (3.1%) of cream cake samples were positive for *Salmonella.* Catering establishments, protective clothing, source of contamination, manner of hand washing, and money handling were among the putative risk factors that were significantly associated (*p* < 0.05) with *Salmonella* spp. occurrence. Ampicillin, nitrofurans, and sulphonamide resistance were significantly associated (*p* < 0.05) with *Salmonella* spp. occurrence in the selected food items. Three (16.7%), 5 (27.8%), 5 (27.8%), and 4 (22.2%) of the isolates were resistant to 3, 4, 5, and 6 antibiotics, respectively, whereas only a sole isolate was resistant to two antibiotics (viz. ampicillin and kanamycin). In conclusion, the general sanitary condition of the catering establishments, utensils used, and personnel hygienic practices were not to the recommended standards in the current study. Besides, detection of multidrug-resistant strains of *Salmonella* in animal origin food items from different catering establishments suggests the need for detailed epidemiological and molecular characterization of the pathogen so as to establish the sources of acquisition of resistant *Salmonella* strains. Hence, implementation of *Salmonella* prevention and control strategies from farm production to consumption of animal origin food items are crucial.

## 1. Introduction

Food-borne diseases occur as a result of consumption of contaminated food stuffs especially from animal products and are a major public health problem globally [1, 2]. With constant changes in the global food trade dynamics, food consumption behaviors, food production environment and processes, and emergence and re-emergence of food-borne pathogens and chemical contaminants entering the food chain, food-borne diseases continue to be a growing problem. An estimated 600 million people, almost 1 in 10 people in the world, fall ill annually for consuming contaminated food and two million deaths are reported each year [3–5]. In developed countries, an estimated one-third of the population is affected by food-borne diseases each year [6]. However, the severity is higher among developing countries including Ethiopia due to poor food handling and sanitation practices, inadequate food safety laws, weak regulatory systems, lack of financial resources, and awareness about proper food handling which creates a conducive environment for the spread of food-borne and food poisoning etiologic agents [7, 8].

Biological contaminants, largely bacteria, constitute the major cause of food-borne diseases [9, 10]. Among the bacteria, *Salmonella* spp. are considered the most prevalent food-borne pathogen that has gained increased attention worldwide in recent years [11] and has long been recognized as an important food-borne disease of economic significance in animals and humans [12], in both developing and developed countries, although incidence rates vary according to the country [13]. *Salmonella* is among common food-borne pathogens predominantly found in beef, poultry meat, pork, eggs, and raw dairy products, acquired directly or indirectly from human or animal excreta [8, 14, 15]. In many countries, high incidence of salmonellosis in man appears to be caused by infection derived from contaminated animal products mentioned above. The contaminated products cause disease as a result of inadequate cooking or cross-contamination of working surfaces in kitchen environment [16, 17].

In Ethiopia, a country in a developing sub-region that experiences the second highest food-borne disease burden in the world [18], the incidence of food-borne *Salmonella* infections has increased dramatically during the past few years. This might be due to unhygienic slaughter practices in the abattoirs, and widespread consumption of raw meat (*Kitfo*, *Kurt*, and *Dulet*) and traditional practices are potential factors contributing to the risk of exposure of the Ethiopian community to food-borne pathogens [1]. Studies conducted in different parts of the country have demonstrated the presence of *Salmonella* in different food animals and food products [13, 14, 18–21]. Despite these attempts to report the prevalence and distribution of *Salmonella* spp. in some animal origin food items, humans, and food animals in Ethiopia, the problem of this pathogen in foods of animal origin is still not well known. However, studies made elsewhere indicated that foods of animal origin are important sources of *Salmonella,* particularly among those raw food consumers.

The emergence of antimicrobial-resistant (AMR) in *Salmonella* is linked with horizontal gene transfer and these genes are found on mobile genetic elements. The expansions of antibiotic-resistant *Salmonella* serovars are efficient in worldwide dissemination [22, 23]. In recent years, AR *Salmonella* has become very common in clinical isolates of animal origin food items [8, 11, 19, 24]. An increase in the resistance of *Salmonella* to commonly used antimicrobials has also been noted in both public health and veterinary sectors in Ethiopia [11, 13, 14, 19–21, 24, 25]. *Salmonella* strains resistant to various antimicrobial agents, particularly resistant to fluoroquinolones and third-generation cephalosporins, are considered as an emerging problem worldwide [26], resulting in higher morbidity and mortality rates and higher overall treatment costs. This may represent a public health risk by transfer of resistant *Salmonella* strains to humans through the consumption of contaminated food and food products.

Ease of access to and high frequency of antibiotic use, use of antibiotics at subtherapeutic levels, over-prescription at health facilities, close contact between animals, high antibiotic use in animals in small production systems, and contamination during handling animal products were among the several factors contributing to high antibiotic resistance in Ethiopia [27]. A recent study revealed very high multidrug resistance to more than two antimicrobial agents in isolates of *Salmonella* from food animals and in contact humans in Ethiopia and elsewhere including resistance to fluoroquinolones and third-generation cephalosporins [28–30].

Therefore, the absence of recent study on the isolation, identification, and antimicrobial resistance profile of *Salmonella* from food of animal origin purchased from different catering establishment in Arsi zone multifaceted with increased consumption of raw/minced meat (locally known as *“Kitfo,” “Dulet,”* and *“Kurt”*), raw milk, egg sandwich and cream cake by the community at large entails isolation, identification, and antimicrobial profile characterization of *Salmonella* food-borne infections that could complement the paucity of information in the selected study setting.

## 2. Materials and Methods

### 2.1. Study Areas

**Figure 1:**
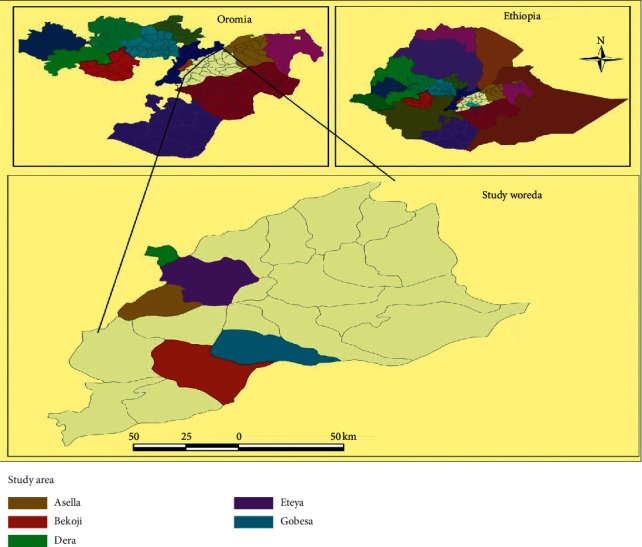
A map that shows study areas.

The study was conducted in Dera, Iteya, Asella, Bekoji, and Gobessa towns in different catering establishments from October 2018 to May 2019 in Arsi Zone of Oromia regional state, southeastern Ethiopia (Figure 1). Dera is the administrative center of Dodota district and has a latitude and longitude of 08°20′N 39°19′E. The altitude of this town ranges from 1400 to 2500 meters above sea level [31], whereas Iteya, the administrative center of Hetossa district, is a town which is located east of Lake Zway with a latitude and longitude of 08°08′N 39°14′ and an elevation of 2215 masl [32]. Asella is located at a distance of 175 km southeast of Addis Ababa at 7°57′N and 39°7′E with an altitude of 1650 to 4130 masl. The annual rainfall of the study area ranges from 200 to 400 mm with mean annual temperature of 22.5°C. The town is with about 367,269 human population inhabitants [33].

Bekoji is a town located at about 235 km southeast of the capital Addis Ababa on the highway towards Bale zone and known as the home town of many famous Ethiopian athletes. The town is the administrative center of Lemu-Bilbilo district located at the latitude and longitude of 7°35′N 39°10′E with an elevation of 2810 m. The area receives an annual rainfall of around 1100 mm with the average annual temperature ranges from 6 to 26^o^C. The human population of the town is estimated at 214, 000 inhabitants [34]. On the same way, Gobessa town is located 265 km southeast of Addis Ababa. Its altitude is between 1500 and 3400 m with an average altitude of 2450 masl. The average temperature is 18°C which varies between 10°C and 25°C, with an annual average rainfall of around 1000 mm. The town is the administrative center of Shirka district with estimated 183,823 human population inhabitants. In the above towns, a number of people are involved in the public service business, including smallholder farming supplying food animals and animal products to the communities and the slaughterhouse [35].

### 2.2. Study Design

A cross-sectional study was conducted from October 2018 to May 2019 to isolate, identify, and characterize antimicrobial susceptibility profiles *Salmonella* spp. from selected animal origin food items in selected towns of Arsi Zone and its suburbs.

Stratified random sampling from catering establishments and a list of frame of public places were used as sample source according to the accessibility of animal origin food items. First, functional catering establishments registered in the towns were searched for and then public places were stratified into strata (hotels, restaurants, cafeterias, and retail shops) and they were used as a sampling frame. Study foods of animal origin were selected during dining time using a simple random sampling method in each public place on proportional bases/considering the availability of the food items.

### 2.3. Sample Size and Study Methodology

Selected animal origin food samples were purchased from hotels, restaurants and cafeterias including minced meat (locally known as “*Kitfo”*, *“Kurt,”* and *“Dulet”*), egg sandwich and cream cake whereas raw milk was bought from retail shops/cafeteria. Time of sampling was scheduled at the beginning of the serving period (breakfast or lunch time).

The sample size calculation was based on 50% prevalence assumption (since there was no study on *E. coli* O157 : H7 and *Salmonella* spp. from different animal origin food items in the selected towns of Arsi Zone), 95% CI and df = 0.05 [36]. Though the sample size calculated for *Salmonella* spp. was 384, only 192 samples were processed for bacteriological detection of *Salmonella* because of the limited number of catering establishments in the study settings as per our stratification. Hence, the total sample size was divided for the four (4) food of animal origin that was sampled (viz. 48 samples for each animal origin food was considered to maintain proportionality).

#### 2.3.1. Method of Sample Collection

The selected animal origin food items including minced meat, egg sandwich, and cream cake samples purchased from the catering establishments were put in sterile plastic bags by using sterile forceps and spoons from the eating plate whereas approximately 10 ml of raw milk bought from different cafeteria/retail shops was collected by using sterile screw capped bottle. All the collected samples were properly identified by sample type, date of collection and sources, and immediately transported to the laboratory (Asella Regional Laboratory, Veterinary Microbiology Section) in an ice box with freeze packs under completely sterile conditions for microbiological analysis.

#### 2.3.2. Isolation and Identification of *Salmonella* Species

Standard cultivation method recommended by the International Organization for Standardization (ISO-6579) [37] with Global *Salmonella* Survey (GSS) and WHO guidelines [38] was carried out for isolation and identification of *Salmonella*. Briefly, 25 gm of each food sample was weighed and homogenized in sterile stomacher bag (Stomacher 400R, Seward, England) with 225 mL of pre-enrichment buffered peptone water for 2 minutes. The nonselective pre-enriched sample, from each solid or liquid food sample, was mixed thoroughly and incubated overnight at 37°C. Following incubation, 1 mL of the pre-enrichment broth was transferred to every 10 mL of tetrathionate broth (Muller Kaufmann, Oxoid, England), for the first selective enrichment of *Salmonella* growth while inhibiting other microorganisms, and incubated at 37°C for 24 hours. In addition, 0.1 mL of pre-enrichment broth was added to 10 mL of Rappaport-Vassiliadis (Oxoid, England) broth for second selective enrichment and incubated at 41°C for 24 hours.

For isolation of *Salmonella* species, each suspected colony from selectively enriched medium was streaked onto selective xylose lysine deoxycholate (XLD) agar plates and incubated at 37°C for 18–24 hours. *Salmonella* suspected colonies, red color with a black center, from XLD agar medium were plated onto nutrient agar (Oxoid, England) for further biochemical confirmation. Biochemical tests: *Salmonella* isolates were identified using triple sugar iron agar (TSI), lysine iron agar, urea broth, indole test, and citrate utilization tests. These were incubated for 24 hours to 48 hours at 37°C. Colonies producing an alkaline slant with acid bottom and hydrogen sulfide production on TSI, positive for lysine, negative for urea hydrolysis, negative for indole test, and positive for citrate utilization were considered as *Salmonella*. Finally, susceptibility to antimicrobial was performed for all isolates.

#### 2.3.3. Antimicrobial Susceptibility Testing for *Salmonella* Species Isolates

Phenotypic antimicrobial susceptibility testing on Mueller–Hinton agar (Oxoid) using the agar disc diffusion method [39] was conducted to determine the antibiotic-resistant profiles of each isolate. Briefly, four well-isolated colonies from nutrient agar plates will be transferred into tubes containing 5 ml of Tryptone soya broth (Oxoid, England). The broth culture was incubated at 37°C for 4 hours until it achieved the 0.5 McFarland turbidity standards. Sterile cotton swabs were dipped into the suspension, rotated several times, pressing firmly on the inside wall of the tube above the fluid level to remove excess inoculums, and swabbed uniformly over the surface of Muller Hinton agar plate (Oxoid, England). The plates were held at room temperature for 30 minutes to allow drying.

Subsequently, the selected antibiotics were placed 15–20 mm apart from each other using sterile forceps to prevent overlapping of the inhibition zones and then, incubated at 37°C for 18 hours. A total of 13 selected antibiotic discs (Oxoid, UK) were included: ampicillin (10 *µ*g), amoxicillin-clavulanic acid (30 *µ*g), gentamicin (10 *µ*g), kanamycin (30 *µ*g), ciprofloxacin (5 *µ*g), chloramphenicol (30 *µ*g), trimethoprim-sulfamethoxazole (5 *µ*g), sulphonamides (300 *µ*g), tetracycline (30 *µ*g), nalidixic acid (30 *µ*g), ceftriaxone (30 *µ*g), streptomycin (10 *µ*g), and nitrofurans (50 *µ*g) (Table 1). Then, the plates were incubated at 37°C for 24 hours and finally the zone of inhibition was measured and interpreted as susceptible, intermediate, or resistant categories assigned on the basis of the critical points recommended by Clinical Laboratory and Standard Institute [39].

### 2.4. Data Management and Analysis

Data generated from laboratory investigations were recorded and coded using Microsoft Excel spreadsheet (Microsoft Corporation) and was analyzed using STATA version 14.0 for Windows (Stata Corp. College Station, TX, USA).

The prevalence of *Salmonella* spp. isolated from the selected animal origin food items was calculated as the number of positive (confirmed) samples divided by the total number of samples investigated (processed) in the laboratory. Logistic regression and/or descriptive statistics such as frequency, percentage, and/or proportion were applied to compute the collected data from the selected foods of animal origin and antimicrobial susceptibility test results.

## 3. Results

### 3.1. Overall Prevalence of *Salmonella spp*

*Isolated from Animal Origin Food Items in the Study Settings.* In the current study, an overall prevalence of *Salmonella* spp. isolated from animal origin food items by conventional culture was 9.4% (18/192). A higher prevalence of 19.2% was observed in Gobessa town compared to the rest study settings but there was no significant association between the study sites and *Salmonella* spp. isolated from animal origin food items (Table 2).

### 3.2. A Chi-Square Analysis of the Putative Risk Factors Associated with *Salmonella* Occurrence in the Animal Origin Food Items

A Chi-square analysis revealed that catering establishments, protective clothing, source of contamination, manner of hand washing (*p* < 0.001), and money handling were significantly associated (*p* < 0.05) with *Salmonella* spp. occurrence among the putative risk factors considered during the study as indicated in Table 3.

### 3.3. Multivariable Logistic Regression Analysis of the Putative Risk Factors Associated with *Salmonella* spp. Occurrence in the Animal Origin Food Items

A logistic regression analysis of the putative risk factors indicated that consumers rinsing their hands with water only were more likely to be infected (AOR = 14.203, 95% CI: 3.088, 65.330) by *Salmonella* spp. pathogenic bacteria upon consuming animal origin food items than consumers using detergents and water while washing their hands as illustrated in Table 4.

### 3.4. Occurrence of *Salmonella* in Animal Origin Food Items and Its Antimicrobial Susceptibility

Antimicrobial susceptibility features of 18 *Salmonella* spp. isolated from different animal origin food items were subjected to 13 panels of antimicrobials to evaluate their resistant level. Among the panels of antimicrobial discs subjected to the isolates, ampicillin, nitrofurans, and sulphonamide resistance was significantly associated (*p* < 0.05) with *Salmonella* spp. occurrence in different types of animal origin food items. All (100%) of the isolates obtained from *“Dulet”* were highly resistant to ampicillin, streptomycin, and nitrofurantoin while 6 (85.7%), 4 (57.1%), and 3 (42.8%) of an isolates were resistant to kanamycin, gentamycin, and tetracycline, respectively. However, none of the isolates were resistant to ciprofloxacin, ceftriaxone, amoxicillin-clavulanic acid, nalidixic acid, sulphonamide, and trimethoprim-sulfamethoxazole which were isolated from *“Dulet.”* Of the 4 isolates obtained from *“Kitfo,”* all (100%) of them were highly resistant to ampicillin and streptomycin while all of them were susceptible to gentamycin, ciprofloxacin, ceftriaxone, amoxicillin-clavulanic acid, nalidixic acid, sulphonamide, and trimethoprim-sulfamethoxazole. Pertaining to the 2 isolates obtained from the raw milk, both of them were highly resistant (100%) to ampicillin, streptomycin, kanamycin, and tetracycline whereas both of the isolates subjected to a panel of antimicrobials were highly susceptible (100%) to ciprofloxacin, ceftriaxone, amoxicillin-clavulanic acid, nalidixic acid, sulphonamide, and trimethoprim-sulfamethoxazole. Besides, interestingly, an isolate from egg sandwich was resistant to streptomycin, kanamycin, and nitrofurantoin while an isolate from cream cake was resistant to gentamycin, ampicillin, streptomycin, kanamycin, nitrofurantoin, and tetracycline as described in Table 5.

### 3.5. Multiple Antimicrobial Resistance Profile of *Salmonella* Species Isolated from Animal Origin Food Items

Out of 18 isolates from different animal origin food items in the selected study settings, all (100%) of them were resistant to two or more panels of antibiotic discs. Of the isolates obtained from the current study, 3 (16.7%), 5 (27.8%), 5 (27.8%), and 4 (22.2%) were resistant to three, four, five, and six antibiotics, respectively, whereas only a single isolate was resistant to two antibiotics (viz. ampicillin and kanamycin) as revealed in Table 6.

## 4. Discussion

Studies on the prevalence and antibiotic susceptibility profiles of *Salmonella* isolates from animal origin food items are scarce in Ethiopia. In the current study, the prevalence and the antimicrobial resistance profiles of *Salmonella* spp. isolated from foods of animal origin were evaluated. The study revealed that an overall prevalence rate of *Salmonella* spp. isolated from animal origin food items by conventional culture was 9.4% in the study areas. A higher prevalence rate of 19.2% was observed in Gobessa town compared to the rest study settings but there was no significant association between the study sites and *Salmonella* spp. isolated from animal origin food items.

Despite certain studies conducted on *Salmonella* isolation from animal origin food items in Ethiopia [13, 40–42], there had been no uniformity with respect to the sample type examined; as a result, overall prevalence of *Salmonella* in animal origin foods may not be comparable. Nevertheless, the overall prevalence of *Salmonella* in this study was lower than the report of [43] from selected African countries (19.9%), [44] from Chinese food commodities (15.3%) and [45] from Thailand retail foods (61%), but higher than the reports of [13] from Gonder, Ethiopia (5.5%), [46] from Morocco (0.91%), and [47] from Lesotho (0.72%). This variation could be due to the differences in the hygienic and sanitary practices of different catering establishments, equipment's used, method of cooking/boiling, the sample type, sampling procedures, and the detection methods employed in different studies.

A higher prevalence rate of *Salmonella* spp. (28.6%) that had been isolated from animal origin food items was sampled from hotel among the catering establishments considered during the study period. This might be due to the limited sample size collected from hotels or the probability of food contamination in this catering was high due to poor sanitary conditions of the establishment and improper handling practice of food. Thus, health hazards from catering establishments may be minimized by avoiding poor handling and awareness of personal hygiene and care in preparation, storage, and dispensing of foods in all procedures necessary to maintain the safety and suitability of food from the establishments, appropriate solid and liquid waste collection and disposal should be planned and implemented and periodic sanitary-hygienic evaluation, and inspection of catering establishments should be strengthened to reduce public health hazards associated with food-borne pathogens using systems such as HACCP [48].

Though most of the food handlers/servants used protective clothes during dinning time, about 15.3% of *Salmonella* isolates were obtained from the samples handled by servants using protective clothing which is inconsistent with the report of [48]. This needs an indepth study to unveil the factors responsible for the occurrence of the pathogen.

Approximately 25% incidence rate of *Salmonella* observed in the current study from minced meat prepared using dirty cutting knife was higher than that reported from Gondar [11] and Mojo [49], Ethiopia, and slightly lower than the reports from Botswana abattoir [50]. The current high prevalence recorded from minced meat prepared by cutting knife might be due to poor hygienic condition of knives and/or high prevalence of *Salmonella* in meat samples which might act as a source of contamination for the knives.

The odds ratio of being positive for *Salmonella* spp. isolation is 14.203 times higher among food handlers who rinsed their hands with water only compared to those who washed their hands with water and detergents. This finding corroborates with earlier studies conducted elsewhere in Ethiopia [51–53]. Personal hygiene differences of the food handlers might help to explain this discrepancy since majority of the workers in the current investigation settings practiced very poor hand washing. Improvement of food worker hand washing practices, the least costly intervention to implement, is critical to the reduction of food-borne illness [11, 54].

The occurrence of *Salmonella* in the minced meat *(Kitfo, Kurt,* and *Dulet)* in contact with bare handed butcher man in this study was 28.6%, which was higher than an isolate obtained from cashier or service woman/man in contact with the sample in the current study. This is attributed to simultaneous handling of food and money which increases the risk of cross contamination [55].

In the current study, though not significant, the highest prevalence of *Salmonella* was isolated from raw *“Dulet”* (fried meat) (21.9%) as compared to raw *“Kitfo”* (minced meat) and *“Kurt”* (chopped meat consumed with or without *“Berbere”* or *“Mitmita”).* This could be due to no clear division of slaughtering process: stunning, bleeding, skinning, evisceration, hanging, and cutting/deboning. Furthermore, there was no preventive mechanism installed for insects and rodents in abattoir/back yard slaughtering where sheep and goat rumen and reticulum are evacuated and cleaned with running water which is similar with the report of [56, 57] from abattoir and butcher shops of Mekele and Bishoftu towns of Ethiopia, respectively. On top of this, this might be due to the fact that many people in Ethiopia also recognize the richness of liver in nutrients and consume it raw with hot pepper or spices. Thus, it is reasonable to assume that meat, milk, and liver can serve as vehicles for extensive transmission of *Salmonella* to humans [18].

The frequency of isolation of *Salmonella* from raw *“Kitfo”* in this study was 12.5%. This finding is comparable with the report of [41] (12.1%) from retail meat products and [42] (14.4%) from minced beef in Ethiopia. However, it is much lower than the report of [58] (42%) from *“Kitfo,”* a traditional Ethiopian spiced, minced meat dish. In contrary to this, our finding is higher than the studies conducted by [40] (7.9%) and [59] (8%) on minced beef in Ethiopia and it is also much higher than 1.8%, 2.0%, and 5.3% of minced meat reported from USA, England, and Germany, respectively [60]. A relatively higher prevalence rate in this study could be attributed to the mincing process in *“Kitfo”* preparation which guaranteed contamination of new surface areas.

The constant isolation of *Salmonella* from some food establishments in the current study indicated that these establishments could have constant sources of contamination with *Salmonella.* Food handlers, who may be carriers of *Salmonella,* are definite sources of contamination if they are not following basic hygienic principles during food handling. Other possible sources of contamination could be cutting boards, which could harbor *Salmonella*, as they are not cleaned thoroughly and are usually moist. *Salmonellae* may establish a niche in a crack in these woody cutting boards, proliferate, and continue to contaminate whatever comes in contact with the cutting board as indicated by [58] in their study. In this study, observation of the kitchen environments in the food establishments showed that the hygienic condition of the kitchens and the food handlers in most cases was very unsatisfactory.

Among the raw minced meat considered in the present study, low prevalence (9.4%) of *Salmonella* was obtained from chopped *“Kurt”* with or without *“Berbere”* (Ethiopian seasoning prepared from dry red chili peppers, garlic, and other spices) or *“Mitmita”* (Bird's eye red pepper spiced with cardamom and salt). The traditional way of eating these foods with fingers might contribute to the contamination of the foods with *Salmonella*. In the category of egg Sandwich, we recorded a contamination rate of 3.1% which is inconsistent with the report of [13] from Gondar, in which no isolate was recorded.

*Salmonella* detection from raw milk (6.3%) in this study was comparable with a study conducted in Nigeria [61] and lower than 20% prevalence rate in Ethiopia [62]. This difference might arise from milking contamination, unclean equipment, and poor hygiene of milkmaid and handlers. However, it is higher than the prevalence of 2.1%, which is found in Ethiopia [63]. As indicated by [64], unclean environmental conditions and poor udder preparation might expose raw milk to bacterial contamination.

Custard cake products in Ethiopia are usually called cream cakes possibly due to the creamy nature of the custard. Though there are large numbers of custard cake consumers among the urban community in Ethiopia, the safety and quality of these products have rarely been assessed. Only a single (3.1%) *Salmonella* isolate was obtained from the cream cakes considered in this work, which is inconsistent with the report of [13] in which no isolate was recorded. A study conducted by [65] revealed that pastry products were among the major food vehicles for outbreaks of *Salmonella* spp. and were responsible for 16.6% of outbreaks in Rio Grande do Sul state, during the periods of 1997 to 1999. The frequency of isolation of *Salmonella* from cream cake (custard cake) in the current study was lower than that recorded in Addis Ababa (10%) [66]. Contamination of custard cakes with *Salmonella* might have arisen from contaminated eggs used for custard preparation and/or *Salmonella* carriers among food handlers. Since custard provides a fertile growth medium, keeping custard cakes for several hours at ambient temperature may support the growth of Salmonellae.

Resistance for two or more of antimicrobials (100%) which was observed in this study was higher than other studies conducted in Ethiopia [59, 67, 68] and elsewhere in the world [69, 70]. This difference may be due to the increasing rate of inappropriate utilization of antibiotics in farm animals which favors selection pressure that increased the advantage of maintaining resistance genes in bacteria [28]. In recent times, the frequency of antimicrobial drug resistance in *Salmonella* and the number of drugs to which the strains are resistant have increased worldwide, primarily as a consequence of antimicrobial use in food production. Recent reports have also highlighted the emergence of *Salmonella* with reduced susceptibility to fluoroquinolones and other drugs [26], which is associated with treatment failures and poor outcomes in human infections.

Zewdu and Cornelius [59] reported that the isolates of *Salmonella* from food items and personnel from Addis Ababa were resistant to the commonly used antibiotics including streptomycin, ampicillin, and tetracycline. The result of the current research also indicated resistance of *Salmonella* isolates to commonly used antimicrobials including streptomycin, ampicillin, nitrofurantoin, kanamycin, and tetracycline, with resistance rate of 100%, 94.4%, 77.8%, 77.8%, and 44.4%, respectively. *Salmonella* resistance in this finding is higher than the previous studies conducted in Ethiopia [59, 62], and other countries [71, 72]. The remarkable rise in the occurrence of antimicrobial resistance in *Salmonella* for the mentioned antibiotics was probably an indication of their frequent usage both in livestock and in public health sectors in Ethiopia. Studies conducted elsewhere in Ethiopia [73] have also indicated that the increase in the proportion of drug-resistant *Salmonella* isolates could be due to the irrational use of antimicrobials and inappropriateness of the prescription and dispensing methods in both the public veterinary and private health setups of the country [13].

In the current study, all of the isolates obtained from different animal origin food items (100%) were resistant streptomycin which is consistent with the report of [18] from bovine or ovine isolates of *Salmonella* in Ethiopia. Thus, it is of significant concern, since our study involves all randomly selected samples. Resistance of *Salmonella* from food items, animals, and humans to streptomycin was reported by several studies in Ethiopia [14, 59, 67, 74, 75] but the level of resistance to streptomycin ranged from 46 to 86%. This extremely high level of resistance of *Salmonella* and other pathogens (e.g., similar high level resistance to streptomycin has also been reported in Ethiopian tuberculosis patients, including in newly diagnosed patients [76] and references therein) to streptomycin in Ethiopia should be cause for high concern as it might also cause cross resistance to other drugs with similar mechanism of action.

Due to the relatively limited access and high price to get the newly developed cephalosporin and quinolone drugs, the reports of prevalence of antimicrobial-resistant *Salmonella* to relatively low-priced and regularly available antibiotics are alarming for a low-income society living in most developing countries, like Ethiopia. However, it is important to note that these antibiotics are commonly used in veterinary medicine, and infections with these resistant *Salmonella* isolates could lower the efficiency of antibiotic treatment [18].

## 5. Conclusion

The current study revealed that *Salmonella* isolates were identified from animal origin food items, viz. raw/minced meat (“*Kitfo*”, *“Kurt,”* and *“Dulet”*), raw milk, egg sandwich, and cream cake, in the selected areas of Arsi zone. Catering establishments, protective clothing, source of contamination, manner of hand washing, and money handling were among the putative risk factors that were significantly associated with *Salmonella* spp. incidence in the selected food items. A high proportion of *Salmonella* isolates resistant to two or more panels of antimicrobials in this study implies a significant public health risk of consuming raw/minced animal origin food items-associated with salmonellosis occurrence. In conclusion, the general sanitary condition of the catering establishments, utensils used, and personnel hygienic practices were not to the recommended standards in the current study. Besides, detection of multidrug-resistant strains of *Salmonella* in animal origin food items from different catering establishments suggests the need for detailed epidemiological and molecular characterization of the pathogen so as to establish the sources of acquisition of resistant *Salmonella* strains. Hence, implementation of *Salmonella* prevention and control strategies from farm production to consumption of animal origin food items are crucial.

## Figures and Tables

**Table 1:** Performance standards for antimicrobial susceptibility testing of *Salmonella*.

No	Antimicrobial agent	Disc code	Potency (*µ*g)	Resistant	Intermediate	Susceptible
1	Ampicillin	AMP	10	≤13	14–16	≥17
2	Amoxicillin-clavulanic acid	AMC	30	≤13	14–17	≥18
3	Ceftriaxone	CRO	30	≤19	20–22	≥23
4	Chloramphenicol	CHL	30	≤12	13–17	≥18
5	Ciprofloxacin	CIP	5	≤20	21–30	≥31
6	Gentamicin	GN	10	≤12	13–14	≥15
7	Kanamycin	KAN	30	≤13	14–17	≥18
8	Nalidixic acid	NA	30	≤13	14–18	≥19
9	Streptomycin	STR	10	≤11	12–14	≥15
10	Tetracycline	TTC	30	≤11	12–14	≥15
11	Trimethoprim-sulfamethoxazole	W	5	≤10	11–15	≥16
12	Sulphonamides	S3	300	≤12	13–16	≥17
13	Nitrofurantoin	NTR	50	≤14	15–16	≥17

Source: CLSI, (2016).

**Table 2:** Overall prevalence of Salmonella isolated from selected type of animal origin food items in the study settings.

Variable category	Number of sample tested	Result of tested sample number		*χ*2 (*p* value)
		Negative sample N (%)	Positive sample N (%)	
Study settings				7.261 (0.117)
Dera	36	32 (88.9)	4 (11.1)	
Iteya	31	31 (100)	0 (0)	
Asella	62	55 (88.7)	7 (11.3)	
Bekoji	37	35 (94.6)	2 (5.4)	
Gobessa	26	21 (80.8)	5 (19.2)	
Total	192	174 (90.6)	18 (9.4)	

*χ*2, Pearson chi-square; N, Number of samples.

**Table 3:** A Chi-square analysis of association of the putative risk factors associated with *Salmonella* spp. prevalence in the animal origin food items in the selected study settings.

Variables category	Number tested	N (%)	*χ* ^2^ (*p* value)
*Sample type*			9.563 (0.096)
Kitfo	32	4 (12.5)	
Kurt	32	3 (9.4)	
Dulet	32	7 (21.9)	
Egg sandwich	32	1 (3.1)	
Raw milk	32	2 (6.3)	
Cream cake	32	1 (3.1)	

*Catering establishments*			10.469 (0.016^*∗*^)
Hotel	14	4 (28.6)	
Restaurant	82	10 (12.2)	
Cafeteria	87	3 (3.45)	
Retail shop	9	1 (11.1)	

*Protective clothing*			8.288 (0.004^*∗*^)
Used	98	15 (15.3)	
Not used	94	3 (3.2)	

*Source of contamination*			6.539 (0.032^*∗*^)
Unclean cutting board#	22	4 (18.2)	
Dirty knife#	12	3 (25)	
Handling with unclean equipment and hands	158	11 (6.9)	

*Manner of hand washing*			13.834 (0.000^*∗∗*^)
Rinsing with water only	41	10 (24.4)	
Using detergents and water	151	8 (5.3)	

*Money handling*			8.019 (0.020^*∗*^)
Butcher with bare hand	14	4 (28.6)	
Cashier	11	2 (18.2)	
Service woman/man	167	12 (7.2)	

*Cutting table (board) available*			6.320 (0.053)
Single for minced meat	85	12 (14.1)	
Separate for minced meat	11	2 (18.2)	
Not available	96	4 (4.2)	

*Origin of the sample*			7.261 (0.117)
Dera	36	4 (11.1)	
Iteya	31	0	
Asella	62	7 (11.3)	
Bekoji	37	2 (5.4)	
Gobessa	26	5 (19.2)	

#, for minced meat; N, number of isolates; ^*∗*^, statistically significant; ^*∗∗*^, statistically highly significant.

**Table 4:** Multivariable logistic regression analysis of putative risk factors associated with *Salmonella* occurrence in animal origin food in the selected study areas.

Variables category	Number of tested samples	Number of positive samples N (%)	Odds ratio		*p* value
			COR (95% CI)	AOR (95% CI)	
*Catering establishments*					0.059
Hotel	14	4 (28.6)	R	R	
Restaurant	82	10 (12.1)	3.2 (0.296, 34.588)	5.048 (0.274, 93.105)	
Cafeteria	87	3 (3.45)	1.1 (0.125, 9.844)	4.674 (0.300, 72.868)	
Retail shop	9	1 (11.1)	0.286 (0.027, 3.076)	0.348 (0.025, 4.772)	

*Protective clothing*					0.010^*∗*^
Used	98	15 (15.3)	5.482 (1.532, 19.614)	7.171 (1.603, 32.083)	
Not used	94	3 (3.2)	R	R	

*Source of contamination*					0.551
Unclean cutting board	22	4 (18.2)	R	R	
Dirty knife	12	3 (25)	2.970 (0.855, 10.310)	0.559 (0.094, 3.332)	
Handling with unclean equipment and hands	158	11 (6.9)	4.455 (1.052, 18.861)	1.846 (0.325, 10.492)	

*Manner of hand washing*					0.001^*∗∗*^
Using detergents and water	41	10 (24.4)	R	R	
Rinsing with water only	151	8 (5.3)	5.766 (2.105, 15.792)	14.203 (3.088, 65.330)	

*Money handling*					0.990
Butcher with bare hand	14	4 (28.6)	R	R	
Cashier	11	2 (18.2)	5.167 (1.408, 18.954)	0.998 (0.151, 6.479)	
Service woman/man	167	12 (7.2)	2.870 (0.556, 14.810)	1.016 (0.099, 10.418)	

AOR, adjusted odds ratio; COR, crude odds ratio; CI, confidence interval; *R*, reference.

**Table 5:** The general proportion of antimicrobial susceptibility test among *Salmonella* isolates from the selected animal origin food items in the study sites.

Antimicrobials tested	Types of sample tested							*χ*2 (*p*-value)
	Kitfo (%) *N* = 4	Kurt (%) *N* = 3	Dulet (%) *N* = 7	Egg Sandwich (%) *N* = 1	Raw milk (%) *N* = 2		Cream cake (%) *N* = 1	
	*n* = 32 for all samples							
*Gentamycin*								20.730 (0.102)
Sensitive	4 (100)	1 (33.3)	2 (28.6)	1 (100)	1 (50)	0		
Intermediate	0	0	1 (14.3)	0	0	0		
Resistant	0	2 (66.7)	4 (57.1)	0	1 (50)	1 (100)		

*Ciprofloxacin*								9.563 (0.093)
Sensitive	4 (100)	3 (100)	7 (100)	1 (100)	2 (100)	1 (100)		
Intermediate	0	0	0	0	0	0		
Resistant	0	0	0	0	0	0		

*Ceftriaxone*								9.563 (0.093)
Sensitive	4 (100)	3 (100)	7 (100)	1 (100)	2 (100)	1 (100)		
Intermediate	0	0	0	0	0	0		
Resistant	0	0	0	0	0	0		

*Ampicillin*								16.779 (0.038^*∗*^)
Sensitive	0	0	0	0	0	0		
Intermediate	0	0	0	1 (100)	0	0		
Resistant	4 (100)	3 (100)	7 (100)	0 (0)	2 (100)	1 (100)		

*Streptomycin*								9.563 (0.093)
Sensitive	0	0	0	0	0	0		
Intermediate	0	0	0	0	0	0		
Resistant	4 (100)	3 (100)	7 (100)	1 (100)	2 (100)	1 (100)		

**Table 6:** Multiple antimicrobial resistance profile of *Salmonella* isolates from animal origin food items in the selected catering establishments of the study areas.

Number of antimicrobial resistance	Antimicrobial resistance patterns (#)	Number of isolates (%) (*n* = 18)
Two	AMP, KAN (1)	1 (5.6)
Three	AMP, STR, KAN (1)	3 (16.7)
	AMP, STR, NTR (1)	
	STR, KAN, NTR (1)	

Four	AMP, STR, NTR, TTC (1)	5 (27.8)
	GN, AMP, STR, KAN (1)	
	GN, AMP, STR, NTR (1)	
	AMP, STR, KAN, NTR (2)	

Five	GN, AMP, STR, KAN, NTR (2)	5 (27.8)
	AMP, STR, KAN, NTR, TTC (2)	
	GN, AMP, STR, KAN, TTC (1)	

Six	GN, AMP, STR, KAN, NTR, TTC (3)	4 (22.2)
	AMP, STR, KAN, NTR, TTC, CHL (1)	

Total		**18 (100)**

GN, gentamycin; AMP, ampicillin; KAN, kanamycin; STR, streptomycin; NTR, nitrofurans; TTC, tetracycline; CHL, chloramphenicol; #, number of isolates which is resistant to a group of antibiotics.

## Data Availability

The data used to support the findings of this study are available from the corresponding author upon request.

## Supplementary Materials

Supplementary Materials All the isolates obtained from the selected food of animal origin were confirmed primarily with the help of *Salmonella* spp. growth and morphological characteristics on xylose lysine deoxycholate (XLD) (Figure S1) and then with biochemical tests (Figures S2–S7) and antimicrobial susceptibility test (Figure S8).
