# Supplementary material for: Occurrence and Antimicrobial Susceptibility Profile of Salmonella Isolates from Animal Origin Food Items in Selected Areas of Arsi Zone, Southeastern Ethiopia, 2018/19
Source: Int J Microbiol. 2021 Mar 31;2021:6633522. doi: 10.1155/2021/6633522 (PMC8026285; doi:10.1155/2021/6633522)
Supplement: Supplementary Materials — All the isolates obtained from the selected food of animal origin were confirmed primarily with the help of Salmonella spp. growth and morphological characteristics on xylose lysine deoxycholate (XLD) (Figure S1) and then with biochemical tests (Figures S2–S7) and antimicrobial susceptibility test (Figure S8). [file 6633522.f1.docx]

**Supplementary Materials**

Supplementary figures showing *Salmonella* colony grown on Xylose Lysine Deoxycholate (XLD) and Brilliant Green agars, different biochemical reaction and drug sensitivity tests results were indicated in supplementary figures below.


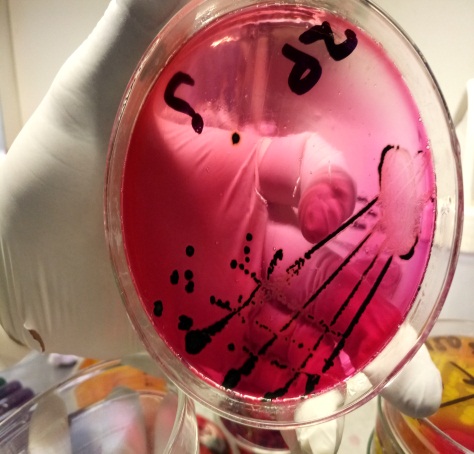


Figure S1 : *Salmonella* colonies grown on Xylose Lysine Deoxycholate (XLD) agar, red color with black centre, from samples of different animal origin food items


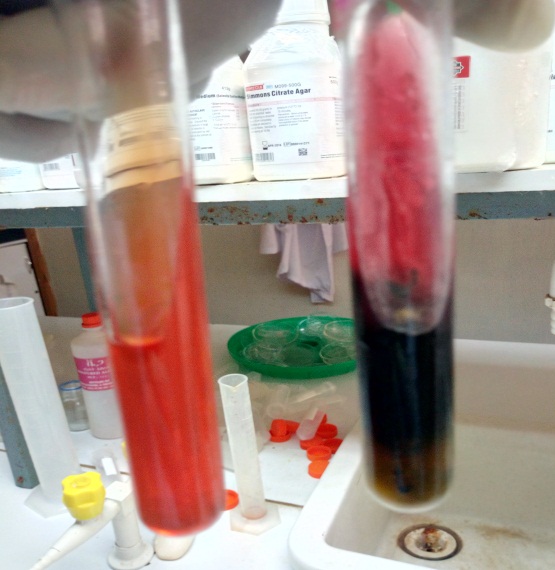


b

a

Figure S2: Triple Sugar Iron (TSI) test (a = uninoculated; b = Postive, Lactose and/or Sucrose fermentation negative, H2S production and gas production positive)


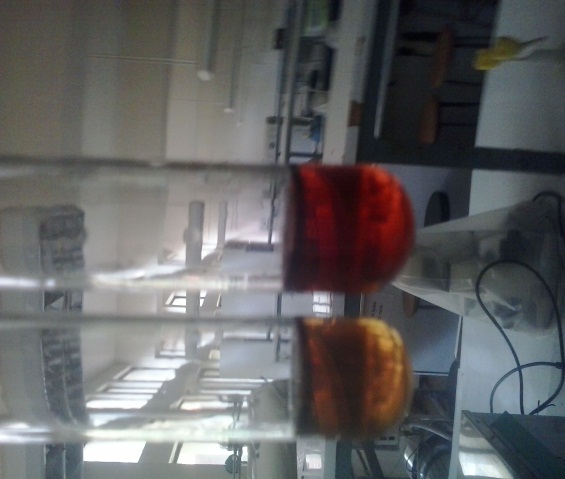


b

a

Figure S3: Voges Proskauer (VP) test (a = Negative; b= Positive (Pinkish color))


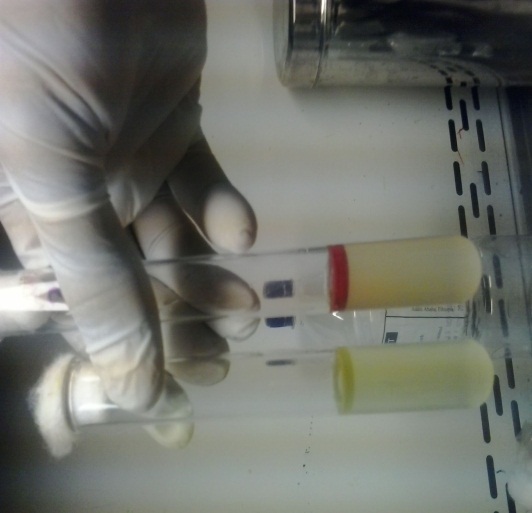


b

a

Figure S4: Indole test ( a= Indole negative, *Salmonella* colony characteristics; b= Indole positive)


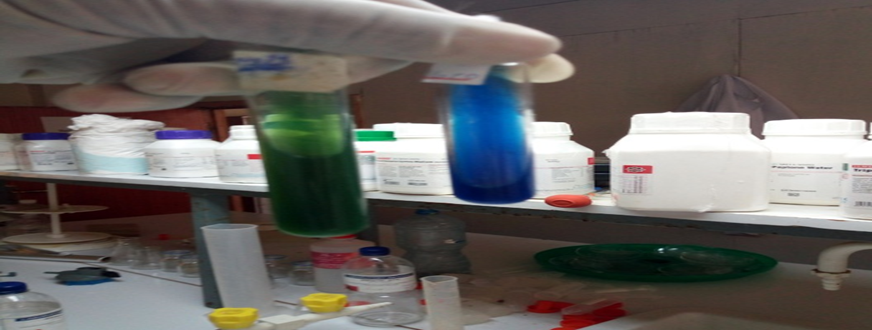


b

a

Figure S5: Citrate utilization test ( a= uninoculated; b= positive, the medium develops blue color from green)


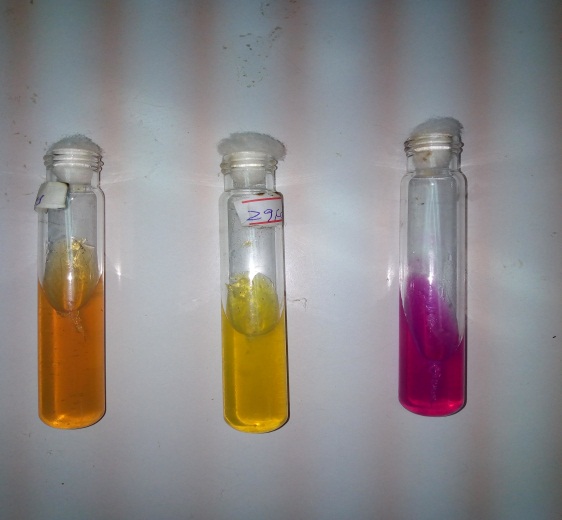


c

a

b

Figure S6: Urea broth (a= uninoculated (control), b = urease negative, *Salmonella* colony characteristics; c= urease positive)


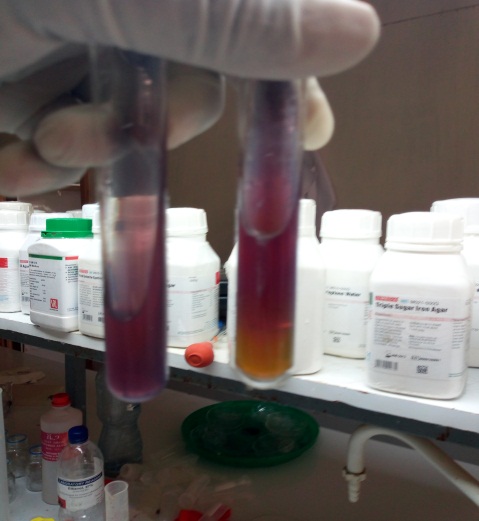


b

a

Figure S7: L-Lysine decarboxylation medium (a= Lysine decarboxylation and H2S production positive and b=Lysine decarboxylation and H2S production negative)


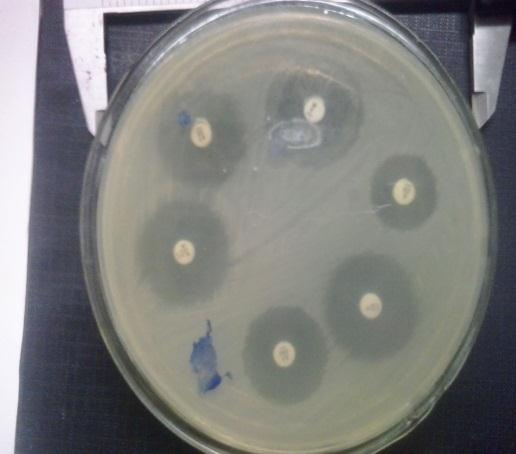

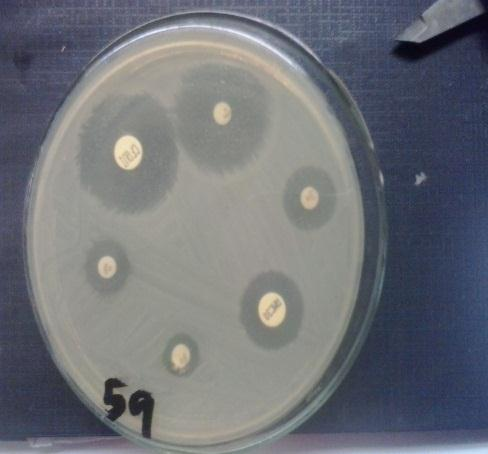


Figure S8: Antimicrobial susceptibility test result for *Salmonella* isolates based on the inhibition zone using the Kirby-Bauer disk diffusion method
